# A Patient with Fatal Necrotizing Fasciitis following the Use of Intra-Articular Sodium Hyaluronate Injections: A Case Report

**DOI:** 10.1155/2013/531794

**Published:** 2013-12-22

**Authors:** Shanti Virupannavar, Carla Guggenheim

**Affiliations:** ^1^Internal Medicine, Michigan State University, 138 Service Road, A225 Clinical Center, East Lansing, MI 48824, USA; ^2^Internal Medicine and Rheumatology, Michigan State University, 138 Service Road, A225 Clinical Center, East Lansing, MI 48824, USA

## Abstract

*Introduction.* Osteoarthritis, a degenerative joint disease, is a key cause of disability around the world and an ever-growing public health concern. Intra-articular hyaluronic acid viscosupplementation is used as a conservative option for osteoarthritis knee pain relief (McArthur et al., 2012; Hootman and Helmick, 2006; Huang el al., 2011). In general, the literature has shown an excellent safety profile for this treatment modality (McArthur et al., 2012; Clegg et al., 2013; Hammesfahr et al., 2003; Neustadt et al., 2005; Cohen et al., 2008; Neustadt, 2003; Jüni et al., 2007; Peterson and Hodler, 2011). *Case Presentation*. In this report, we describe a case of a woman who had received multiple sodium hyaluronate injections and developed severe necrotizing fasciitis near the injection site. *Conclusion.* We recommend that clear guidelines for clean technique be put in place for use with sodium hyaluronate injections and consideration of full sterile technique in immunosuppressed patients.

## 1. Introduction

Osteoarthritis (OA), a degenerative joint disease, is a key cause of disability around the world and a large, ever-growing public health concern. Both obesity and the aging population are expected to increase the prevalence of OA [1, 2]. Sixty-seven million Americans, about 25% of the adult population, are projected to have OA by 2030 [2]. OA is characterized by progressive cartilage damage and loss of the synovial fluid's lubricating and viscoelastic properties [3]. Intra-articular hyaluronic acid viscosupplementation was first approved by the Food and Drug Administration (FDA) in 1997 and is used as a conservative option for OA knee pain relief [1–3]. Synovial fluid and articular cartilage contain hyaluronan, a natural compound that reduces the coefficient of friction, thereby giving ease in joint movement [3]. An osteoarthritic knee has up to 50% less hyaluronan and the molecule itself is degraded, reducing its molecular weight. This causes increase in coefficient of friction and difficulty with joint movement [4]. Hyalgan (Fidia, S.p.A., Abano Terme, Italy) is a sodium hyaluronate intra-articular injectable medication, derived from rooster combs, having identical viscoelastic properties as human hyaluronic acid [5].

Viscosupplementation, with intra-articular sodium hyaluronate, is now a widely used method for treatment of knee OA. In general, the literature has shown an excellent safety profile for this treatment modality, with the most common adverse events being mild injection site pain and swelling [1, 4, 6–11]. In this report, we describe a case of a woman who had received a total of 30 sodium hyaluronate injections over the course of 5 years and developed severe necrotizing fasciitis near the injection site.

## 2. Case Presentation

Our patient is a 63-year-old female with osteoarthritis in the lumbar spine and bilateral knees. Knee X-rays showed bilateral mild osteoarthritic changes. MRI of left knee showed grade II-III patellar chondromalacia and mild osteoarthritic changes. Her left knee pain was uncontrolled with conservative measures including NSAIDs and narcotics. Steroid injections were provided once with no pain relief. Subsequently, over a period of five years, she received multiple courses of sodium hyaluronate injections to the left knee without complications, waiting at least six months between each series of five injections. The skin was thoroughly examined prior to each injection and was noted to be in a good condition, with no breaks, rashes, or bruises evident. She had great pain relief from the Hyalgan injections and repeatedly requested them. Clean technique was employed prior to each of these injections, including use of betadine, alcohol, and gloves.

Pertinent past medical history included treatment for invasive ductal left breast cancer with lumpectomy and radiation five years prior to this hospital presentation. She had been in remission until four months prior to hospitalization, when she was diagnosed with recurrent left breast ductal carcinoma. She then underwent mastectomy with axillary lymph node dissection and received one round of chemotherapy treatment, with Taxotere, Carboplatin, and Herceptin, 11 days prior to hospital admission. The surgery and chemotherapy proceeded without complications.

Five days after the last sodium hyaluronate injection to the left knee, she presented to the hospital with complaint of left leg pain that woke her from sleep. She initially noticed a small area of erythema in the left popliteal fossa. Over the next few hours, she developed rapidly progressive swelling, expansion of the erythema, and formation of multiple large bullae extending from the thigh to proximal leg. By the time she arrived at the hospital, the bullae and erythema had expanded to the gluteal fold and inguinal region. CT of left lower extremity showed free gas through the adductor musculature and posterior and superior to ischial tuberosity. Given the aggressive symptoms, the patient was emergently taken to operating room for extensive debridement and amputation at the lesser trochanter. She was then admitted to intensive care unit on broad spectrum antibiotics for septic shock secondary to necrotizing fasciitis of the left lower extremity, involving both posterior and anterior compartments. She deteriorated and required vasopressors, intubation and multiple debridements. Over the next 3 days, she suffered multiorgan failure and required hemodialysis. Given her wishes and poor prognosis, life support was withdrawn. She died 20 days after last chemo, 13 days after the most recent Hyalgan injection, and 7 days after the onset of necrotizing fasciitis.

## 3. Discussion

Necrotizing fasciitis is a rare, but potentially fatal, rapidly progressive soft tissue infection involving subcutaneous fat and fascia [12, 13]. Early diagnosis, use of antibiotics, and debridement are essential [12, 13]. The causative bacteria could be aerobic, anaerobic, or mixed flora [12]. The most common pathogens are streptococci and *S. aureus* [12]. Risk factors include immunosuppression, diabetes, trauma, and operative infections [12]. There are case reports of necrotizing fasciitis occurring after intra-articular corticosteroid injections and diclofenac injections [14–19]. However, there are no previously described cases of necrotizing fasciitis following viscosupplementation. As mentioned earlier, sodium hyaluronate injections have proven to be quite safe and have a low incidence of serious side effects [1, 4, 6–11]. The overall incidence of side effects is approximately 1–3% per injection [20]. The most common side effects include injection site pain, GI complaints, local skin reaction, and pruritis; rare serious adverse outcomes are septic arthritis and gout [6, 20–23].

Apart from the issue of adverse outcomes, the efficacy of viscosupplementation has recently been called into question [24–26]. In 2012, Rutjes et al. did a systematic review of 89 large trials, involving 12,667 patients, and showed that there was only a small, clinically irrelevant benefit in terms of pain, with no positive effect on function [24]. Jørgensen et al. did a multicenter, randomized, and double blinded study of 337 patients and found no improvement in pain or function compared to placebo. The American Association of Orthopedic Surgeons recommends neither for nor against the use of viscosupplemention for the nonarthroplasty treatment of OA of the knee based on their systematic review of the published studies [24, 25, 27].

Typically, patients who develop necrotizing fasciitis have a preexisting condition making them more susceptible to infection [12, 13]. Our patient was recently treated for invasive ductal carcinoma of the left breast, with chemotherapy 11 days prior to hospitalization. She was likely immunosuppressed secondary to malignancy and recent use of chemotherapeutic agents. Necrotizing fasciitis most frequently occurs following local trauma which compromises skin integrity [12, 28]. Our patient's rapidly spreading, fatal necrotizing fasciitis started near the site of her multiple injections. Sodium hyaluronate injections are contraindicated if infection or skin disease is present at the injection site, in order to reduce risk of septic arthritis [5]. However, there is no mention of avoiding viscosupplementation in immunosuppressed patients, such as diabetics or patients with underlying malignancy. Our patient was examined by physician prior to each injection and was noted to have no local skin condition near the injection site.

Though our patient had no skin compromise at the injection site, this possibly was the source of bacterial inoculation. The American College of Rheumatology guidelines state that sterile gloves should be worn for intra-articular steroid injections [29]. However, Charalambous et al., in their survey, noted that 53.4% of responders did not follow aseptic techniques prior to these injections [21]. It should further be noted that there are no proper guidelines regarding implementation of clean or aseptic technique with Hyalgan injections.

## 4. Conclusion

Despite the rarity of adverse events, intra-articular sodium hyaluronate injections can cause life-threatening infections. Furthermore, the long-term effectiveness of these injections is still a matter of debate [24, 25, 30, 31]. We recommend clear guidelines for clean technique be put in place for use with sodium hyaluronate injections. In immunosuppressed patients, it may be necessary to observe full sterile technique, given the increased likelihood of infection. Further studies should be done examining the incidence of adverse events following such injections in immunosuppressed patients. The practical implications of such studies could be crucial, as many patients receiving these injections may be on concomitant immunosuppressive agents such as disease modifying antirheumatic drugs (DMARDS) or chemotherapeutic agents.
